# The posttraumatic cognitive appraisal inventory (PTCAI): development and validation

**DOI:** 10.3389/fpsyg.2023.1224984

**Published:** 2023-12-22

**Authors:** Wenyue Zhang, Wenjing Yu, Baojian Wei, Qianni Dong, Aihua Zhang

**Affiliations:** ^1^School of Nursing, Shandong First Medical University & Shandong Academy of Medical Sciences, Tai'an, Shandong, China; ^2^Tai'an Municipal Hospital, Tai'an, Shandong, China

**Keywords:** accidental trauma, cognitive appraisal, inventory, reliability, validity

## Abstract

**Objective:**

This study aims to develop and validate the Posttraumatic Cognitive Appraisal Inventory (PTCAI) for accidental trauma survivors.

**Method:**

Based on interviews and expert feedback, the initial item pool was generated for the Negative Cognitive Appraisal Inventory of Loss and Feeling Threatened, and the Positive Cognitive Appraisal Inventory of Positively Face, Self-Sense, and Relationships. Then, we recruited two groups of accidental trauma survivors to examine the reliability and validity of the PTCAI. Item analysis and exploratory factor analysis (EFA) were conducted on Sample 1. Confirmatory factor analysis (CFA), Pearson correlation analysis, and internal consistency reliability analysis were applied to Sample 2. After 2 weeks, 20 survivors completed the PTCAI again to test temporal stability.

**Results:**

Following item analysis, the PTCAI was reduced to 27 items. The results of the EFA demonstrated that the five-factor, 27-item solution of the PTCAI was appropriate, which accounted for 63.931% of the total variation. The CFA indicated that the five-factor second-order model offered an excellent fit to the data. Loss and Feeling threatened were equally important in the study participants’ negative cognitive appraisal of accidental traumas. Self-sence was the most important positive cognitive appraisal of accidental traumas by the study participants. Positively Face and Relationships were somewhat behind. Additionally, the PTCAI demonstrated high concurrent validity and reliability (test–retest and internal consistency).

**Conclusion:**

The PTCAI appears to be a reliable and valid instrument for assessing cognitive appraisals of accidental trauma survivors.

## 1 Introduction

About 80% individuals will be exposed to accidental traumas throughout their lives (Frans et al., 2005). According to the World Health Organization (WHO), 4.4 million deaths globally from accidental traumas occurred in 2019 alone, making up 8% of the total number of deaths (World Health Organization, 2022). Accidental traumas can range from a variety of events, including traffic accidents, fall injuries, and work-related injuries, etc., which are characterized by suddenness, unpredictability, and uncontrollability (Mock et al., 2017). Although accidental trauma survivors have negative psychological reactions such as post-traumatic stress disorder (PTSD), recovery or resilience is typically result of accidental trauma survivors (Bonanno et al., 2011). According to Lazarus and Folkman (1984), the cognitive appraisal plays an extensive part in the development of physical and psychological symptoms.

The cognitive appraisal was the process by which people evaluated the nature, severity, and possible harm of the traumatic events they had encountered (Lazarus and Folkman, 1984). It has been proposed that the mental health effects of a stressful situation may depend more on how that event is appraised than on the stressor itself (Carpenter, 2016). The cognitive appraisal is the key intermediate factor in determining whether traumas can cause an individuals’ stress response (Kube et al., 2023). The Diagnostic and Statistical Manual of Mental Disorders Fifth Edition (DSM-5) now clearly recognizes trauma-related cognitive appraisals as diagnostic criteria critical to the onset and course of PTSD (Valdez et al., 2021).

Taylor (1983) believed that even dangerous situations may have a silver lining. Exploring the meaning of life, resolving traumatic events or situations, and rebuilding one’s sense of value might help those who have experienced accidental traumas find a new dynamic equilibrium with the outside world. As time goes by, survivors of accidental traumas tend to rethink their traumatic experiences and the physical and mental disturbances caused by them and try to seek the benefits of the events with a positive attitude. Survivors who go through this cognitive adaptation process have positive cognitive appraisals of accidental traumatic events. Positive and negative cognitive appraisals of accidental traumatic events are not mutually exclusive. An individual can appraise an event with more than one attitude at the same time.

To better understand the cognitive appraisal of posttraumatic survivors, researchers have developed several measures related to posttraumatic cognitive appraisals. Currently, measures that tap into trauma-related cognitions include the Posttraumatic Cognitions Inventory (PTCI) (Foa et al., 1999), the Functional Posttraumatic Cognitions Questionnaire (FPTCQ) (de Haan et al., 2021), the Trauma Appraisal Questionnaire (TAQ) (DePrince et al., 2010), the Cognitive Appraisal of Health Scale (CAHS) (Kessler, 1998), the Illness Cognition Questionnaire (ICQ) (Evers et al., 2001), the Meaning of Illness Questionnaire (MIQ) (Browne et al., 1988), the Trauma-Related Cognitions Scale(TRCS) (Valdez et al., 2021), the Posttraumatic Maladaptive Beliefs Scale (PMBS) (Vogt et al., 2012), the Stress Appraisal Measure (SAM) (Peacock and Wong, 1990), the Primary Appraisal/Secondary Appraisal scale (PASA) (Gaab et al., 2005), etc.

Among these scales, the PTCI consists of three dimensions: negative cognitions about the self, negative cognitions about the world, and self-blame. In the original study, the scale had excellent internal consistency, test–retest reliability, convergent validity, and discriminative validity and has been further validated in a sample of motor vehicle accident (MVA) survivors (Beck et al., 2004). However, the PTCI solely focuses on negative cognitive appraisals of traumatic events, ignoring positive psychological experiences. The FPTCQ is an evaluation tool developed for children and adolescents to assess their posttraumatic cognitions. The questionnaire consists of 11 items and has good reliability and validity (e.g., “I can live with what happened.”). However, this scale has not yet been applied in adults, and all 11 items reflect positive cognitive appraisals of accidental traumas by the participants. Neither PTCI nor FPTCI can fully understand individuals’ cognitive appraisals of accidental traumas.

Several assessment instruments (such as CHAS, ICQ, MIQ and, PTCI et al.) have been validated in patients with chronic illness, cancer, or who have been sexually assaulted may not be fully applicable to patients with accidental traumas. In contrast to traumas arising from non-accidental causes (such as chronic illnesses), accidental traumas are characterised by their sudden and uncontrollable nature and their short duration and cause intense fear, helplessness, and/or the perception that they might die. And patients often suffer temporary or permanent disability and need long-term physical and mental health care and rehabilitation. Moreover, in contrast to the excessive self-blame of patients who have been sexually assaulted, accidental trauma survivors prefer to attribute the trauma to other people, the environment, or chance. Such an attribution implies that they will be protected from blame (Bulman and Wortman, 1977; Beck et al., 2004). Thus, accidental trauma patients may have different cognitive appraisals from other types of traumas such as chronic illnesses. Therefore, it is necessary to develop a systematic and complete assessment tool for accidental trauma survivors, including both negative and positive cognitive appraisals – the Posttraumatic Cognitive Appraisal Inventory (PTCAI).

## 2 Materials and methods

### 2.1 Initial development of the PTCAI

33 items were developed based on detailed clinical interviews with survivors of accidental traumas (e.g., motor vehicle accidents, occupational injuries, fall injuries, and fights) (Yu et al., 2019), as well as scales that were directly related to our study. Following that, the content validity of the 33 items was evaluated by five nursing experts with extensive experience, and professional knowledge in nursing or psychology. They independently evaluated the category of Trauma-related cognitions that each item measured. Then, the evaluators discussed each item in turn to reach a consensus on which category of trauma-related cognitions the item in question measured. Only the most representative items from the draft item pool were kept after ambiguous items (e.g., I had to give up a lot because of the trauma; The trauma happened because of the way I acted.) and items with similar significance (For example, we combined “I value life more” and “I can value each day better” into “I value life more now than I did before the trauma.) were removed. At the same time, we have added three new items according to expert opinions. The first draft of the PTCAI was made up of 33 items including 6 items for Loss, 5 items for Feeling Threatened, 8 items for Positively Face,10 items for Self-Sense, and 4 items for Relationships (see Appendix 1). Loss (e.g., The trauma breaks my current life.) reflects participants’ negative cognitive appraisal of the loss of their physical and mental health or resources as a result of the trauma. Feeling Threatened (e.g., My post-traumatic health condition scares me.) reflects participants’ negative cognitive appraisal of potential loss from trauma. Positively Face (e.g., I can accept the fact that I’m injured very well.) reflects participants’ acceptance of physical and psychological damage caused by the trauma. Self-sence (e.g., The power of my fellow patients’ example reacquires my faith in life.) reflects the positive change in the participant’s view of life and the world after trauma. Relationships (e.g., I have more sympathy for others.) reflects positive changes of attitudes towards family, close friends and relatives in participants after trauma. Each item is given a rating on a 5-point Likert scale, from strongly agree (5) to strongly disagree (1). The PTCAI score for each dimension is the total of the PTCAI scores for each item, and the total score is the total of the PTCAI scores for each dimension.

### 2.2 Participants

For this study, we recruited 437 accidental trauma survivors among inpatients in the orthopaedic and trauma departments of three tertiary-level hospitals in Tai’an City. Participants were randomised into two groups. Individuals qualified for assessment if they had experienced an accidental trauma that resulted in a somatic impairment, are currently in recovery which is the period during which the participants’ physical dysfunction or disability resulting from the accidental trauma is eliminated or mitigated through treatment and training, and are between the ages of 18 and 65. Additionally, we excluded patients with communication disorders caused by aphasia, dysarthria, etc., and survivors of previously diagnosed psychiatric disorders. In Sample 1, 212 questionnaires were distributed. After eliminating invalid questionnaires (response time ≤ 4 min, repetitive or regular response patterns to 10 or more questions and with missing date), 200 valid questionnaires remained—a valid response rate of 94%. The age of the participants ranged from 19 to 65 years (*M* = 42.87, SD =11.36), 68 were women (34%), and 132 were men (66%); 23.5% had a college degree, 32.5% reported having some high school, and 44% reported having a junior high school education or less; 94(47%) suffered work-related injuries, 89(44.5%) were injured in car accidents, and 17(8.5%) suffered other types of accidents. Most patients (50%) are injured for less than 3 months. The majority of patients were injured for less than 3 months, 37% were injured between 3 months and 12 months, and 13% were injured for more than 12 months. 36.5% of patients reported having been admitted to a care unit after their injury. The average score of the Injury Severity Scale (ISS) was 15.02 (SD =8.33), with a range from 6 to 50 points. Sample 2 was studied with 225 accidental trauma survivors. During data pre-processing, we removed invalid questionnaires and the final remaining 220 samples were screened—a valid response rate of 97.8%. The age of the participants ranged from 22 to 65 years (*M* = 42.98, SD =11.44), 73 were women (32.2%), and 147 were men (66.8%); 20.9% had a college degree, 28.6% reported having some high school, and 50.5% reported having a junior high school education or less. 95(43.2%) suffered work-related injuries, 102 (46.4%) were injured in car accidents, and 23 (10.5%) suffered other types of accidents. 45% of patients were injured for less than 3 months, 42.7% were injured between 3 months and 12 months, and 11.8% were injured for more than 12 months. 37.3% of patients reported having been admitted to a care unit after their injury. The average score of the Injury Severity Scale (ISS) was 16.10 (SD =8.97), with a range from 4 to 50 points. Written informed consent was obtained after subjects received a thorough written and verbal explanations of the study.

### 2.3 Measures

#### 2.3.1 The Mos 36-item short form health survey – Chinese version

The Mos 36-item Short Form Health Survey (SF-36) was developed by Ware (1993) to assess the quality of life, which was later translated into Chinese by Li et al. (2002). Eight dimensions of Physical Functioning, Role Physical, Bodily Pain, General Health, Vitality, Social Functioning, Role Emotional, and Mental Health, with 36 items, make up the two subscales of the SF-36 that measure physical and mental health, respectively. Cronbach’s α of all eight dimensions is greater than 0.7. Scores vary from 0 to 100, with higher values reflecting higher quality of life.

#### 2.3.2 The hospital anxiety and depression scale

The Hospital Anxiety and Depression Scale (HADS) was developed by Zigmond and Snaith (1983) and is primarily used to assess anxiety and depression in hospitalized patients. The HADS consists of 14 items, 7 of which evaluate depression and 7 of which evaluate anxiety. The Cronbach’s α for the anxiety and depression subscales are 0.85 and 0.79, respectively.

#### 2.3.3 The posttraumatic growth inventory – Chinese version

The Posttraumatic Growth Inventory (PTGI) was developed by the American scholars Tedeschi and Calhoun (1996) and later translated and revised into Chinese by Wang et al. (2011), mainly to measure the degree of positive psychological change after traumas. The Chinese version has 20 items and 5 dimensions, including Relating to Others, New Possibilities, Personal Strength, Spiritual Change, and Appreciation of Life. Each item is rated on a 6-point Likert scale, ranging from “very much (5)” to “not at all (0),” with higher scores indicating higher levels of posttraumatic growth. The Cronbach’s α for the PTGI is 0.90 and the Cronbach’s α for each dimension ranges from 0.67 to 0.85.

### 2.4 Statistical analysis

The statistical analyses were conducted through SPSS version 20.0 and AMOS version 24.0. First, using SPSS 20.0, we conducted item analysis and exploratory factor analysis (EFA) on Sample 1. Confirmatory factor analysis (CFA) was then applied to further examine the factor structure that suggested by the EFA results in sample 2. Furthermore, the PTCAI was given twice, separated by 2 weeks, to the 20 participants in Sample 2. By doing a Pearson correlation analysis on the two scores, the temporal stability was tested. And, internal consistency was examined by calculating Cronbach’s α of the PTCAI. In addition, for studying concurrent validity, we conducted Pearson correlations for PTCAI, SF-36, HADS, and PTGI.

## 3 Results

### 3.1 Item analysis

To further optimize the items of the scale, item analysis was completed using Sample 1. Items from the PTCAI that did not differ significantly between the groups with high and low scores (*p* > 0.05) were eliminated using the critical ratio method. The 200 scales were sorted by total score, and the critical values of the high group and the low group are 27% (71 points) and 73% (86 points), respectively. The independent samples t-test was then used to test the significance of the differences in mean scores between the high group (top 27%) and the low group (bottom 27%). Items were deemed sufficiently different to be preserved if their 95% confidence intervals did not include 0. The results indicated that items 17, 18, 23, 28, and 33 did not satisfy the requirements. However, item 17 was reserved according to the expert opinion. Additionally, based on the results of the correlation analysis, items 18, 23, 25, 27, 28, and 33, for which the correlation between each item score and the total score was less than 0.4 (*p* > 0.01) were excluded. The results are shown in Table 1. Thus, the PTCAI was reduced for the following analyses to 27 items.

**Table 1:** Item analysis - critical ratio results, and item-total-correlation of the PTCAI items (N = 200).

Item	Mean ± SD	High (*N* = 55)	Low (*N* = 58)	*t*	*p*	Item-total correlation
1	2.96 ± 1.40	4.00 ± 0.78	1.86 ± 0.98	−11.11	<0.01	0.58^**^
2	2.75 ± 1.48	3.66 ± 1.22	1.84 ± 1.24	−6.82	<0.01	0.55^**^
3	2.93 ± 1.17	3.85 ± 0.85	1.93 ± 0.66	−11.65	<0.01	0.66^**^
4	2.99 ± 1.11	3.63 ± 0.89	2.25 ± 0.89	−7.28	<0.01	0.50^**^
5	2.90 ± 1.16	3.95 ± 1.00	2.59 ± 1.06	−6.07	<0.01	0.51^**^
6	3.27 ± 1.16	3.63 ± 0.99	2.07 ± 0.87	−7.73	<0.01	0.54^**^
7	3.17 ± 1.19	3.88 ± 0.87	2.27 ± 1.06	−7.77	<0.01	0.59^**^
8	3.58 ± 1.58	4.15 ± 1.39	2.80 ± 1.36	−4.54	<0.01	0.50^**^
9	2.82 ± 1.13	3.61 ± 1.12	2.09 ± 0.77	−7.25	<0.01	0.50^**^
10	3.58 ± 1.43	3.71 ± 0.78	2.32 ± 0.60	−9.22	<0.01	0.54^**^
11	3.19 ± 0.99	3.78 ± 0.69	2.52 ± 0.85	−7.52	<0.01	0.50^**^
12	3.50 ± 0.93	3.93 ± 0.69	3.05 ± 0.94	−4.97	<0.01	0.46^**^
13	3.29 ± 0.93	3.73 ± 0.78	2.89 ± 0.90	−4.64	<0.01	0.69^**^
14	4.20 ± 0.68	4.49 ± 0.51	4.00 ± 0.68	−3.72	<0.01	0.43^**^
15	3.99 ± 0.71	4.12 ± 0.71	3.61 ± 0.95	−2.81	<0.01	0.56^**^
16	4.09 ± 0.74	4.68 ± 0.47	3.64 ± 1.31	−4.95	<0.01	0.76^**^
17	4.28 ± 0.80	4.61 ± 0.49	4.34 ± 1.03	−1.51	>0.05	0.52^**^
18	2.56 ± 1.54	2.76 ± 1.63	2.55 ± 1.44	−0.63	>0.05	−0.20
19	3.52 ± 0.87	3.83 ± 0.83	3.25 ± 0.89	−3.09	<0.01	0.46^**^
20	3.21 ± 0.84	3.63 ± 0.70	2.89 ± 0.81	−4.53	<0.01	0.41^**^
21	3.83 ± 0.77	4.22 ± 0.61	3.41 ± 0.82	−5.15	<0.01	0.44^**^
22	4.24 ± 0.52	4.44 ± 0.50	4.05 ± 0.53	−3.53	<0.01	0.54^**^
23	4.08 ± 0.44	4.12 ± 0.56	3.93 ± 0.33	−1.89	>0.05	0.16
24	4.66 ± 0.52	4.78 ± 0.48	4.57 ± 0.50	−2.01	<0.05	0.62^**^
25	3.90 ± 0.78	4.32 ± 0.60	3.70 ± 0.82	−3.87	<0.01	0.36^*^
26	4.28 ± 0.45	4.41 ± 0.50	4.18 ± 0.39	−2.39	<0.05	0.41^**^
27	2.80 ± 1.39	3.56 ± 0.38	2.27 ± 1.13	−4.73	<0.01	0.34^*^
28	4.48 ± 0.50	4.49 ± 0.51	4.45 ± 0.50	−0.30	>0.05	0.09
29	3.88 ± 0.97	4.12 ± 0.64	3.43 ± 1.13	−3.50	<0.01	0.56^**^
30	3.84 ± 1.01	4.12 ± 0.64	3.34 ± 1.16	−3.88	<0.01	0.49^**^
31	3.91 ± 1.03	4.20 ± 0.68	3.32 ± 1.14	−4.35	<0.01	0.42^**^
32	4.44 ± 0.64	4.63 ± 0.49	4.3 ± 0.60	−2.86	<0.05	0.40^**^
33	4.08 ± 1.02	4.24 ± 1.09	3.93 ± 1.02	−1.36	>0.05	0.30^*^

^*^Means *p*<0.05; ^**^Means *p*<0.01.

### 3.2 Exploratory factor analysis

Data suitability for such modeling was evaluated before EFA. The size of Sample 1 (*n* = 200) met the requirement of 100 cases (Ferguson and Cox, 1993), which was the bare minimum. Bartlett’s tests of Sphericity, which reached statistical significance, supported the suitability of the correlation matrix for modeling (χ^2^ = 3308.4, *d*ƒ = 351, *p* < 0.001). And the Kaiser-Meyer-Olkin (KMO) met the criterion of greater than 0.5 (Chung et al., 2023) (KMO = 0.731). For Sample 1, a Principal Components factor analysis (PCA) with oblique rotation was carried out. The Scree test and eigenvalues greater than 1 suggested that a five-factor solution was the most appropriate, explaining 63.931% of the total variance (see Table 2). All 27 items met the factor loading criterion of greater than 0.3 (Chung et al., 2023), and therefore all items were retained (see Table 3). The variation was explained by the five factors in turn by amounts of 20.311, 13.157, 12.923, 10.772, and 6.768%, respectively. Examination of the items with high factor loadings indicated that the factors reflected (a) Loss, (b) Positively Face, (c) Relationships, (d) Feeling Threatened, and (e) Self-Sense. The composition of the factors is consistent with the *a priori* allocation of items. Finally, the formal version of the PTCAI scale, consisting of five dimensions and 27 items, was developed (see Appendix 2).

**Table 2:** Eigenvalue and variance explained rate of the PTCAI (N = 200).

Factor	Eigenvalue	Variance explained rate (%)	Cumulative variance explained rate (%)
Loss	5.439	20.311	20.311
Positively Face	3.552	13.157	33.468
Relationships	3.489	12.923	46.391
Feeling Threatened	2.809	10.772	57.163
Self-Sence	1.856	6.768	63.931

**Table 3:** Factor loadings of exploratory factor analysis for the PTCAI (*N* = 200).

Item	Factor				
	Loss	Positively Face	Relationships	Feeling Threatened	Self-Sence
9	0.830				
2	0.813				
5	0.788				
1	0.767				
3	0.741				
4	0.572				
13		0.814			
21		0.801			
12		0.796			
20		0.745			
14		0.661			
19		0.606			
22		0.527			
30			0.96		
29			0.953		
31			0.935		
32			0.611		
8				0.953	
10				0.932	
6				0.856	
11				0.696	
7				0.685	
15					0.798
16					0.742
26					0.538
17					0.474
24					0.444

### 3.3 Confirmatory factor analysis

**Figure 1:**
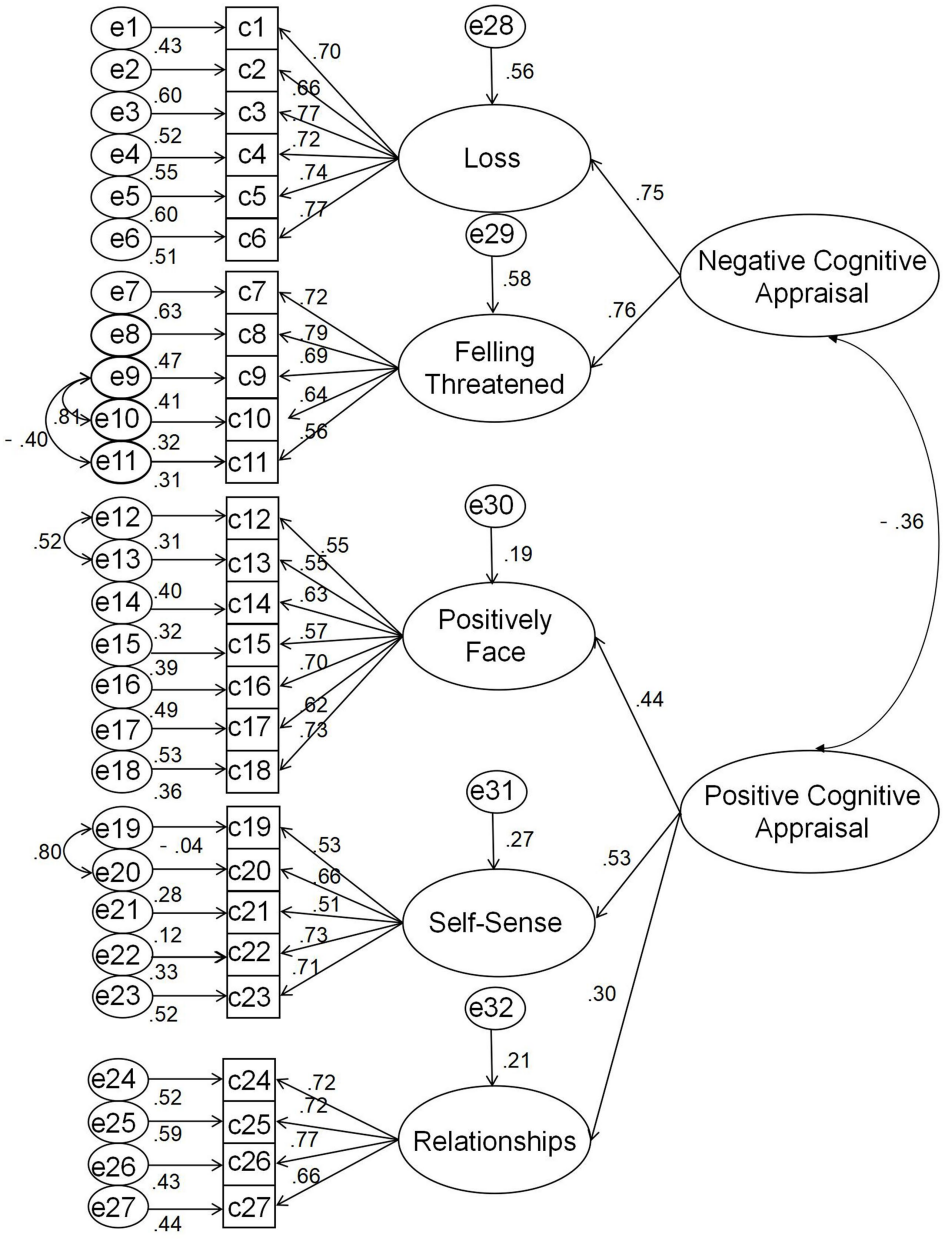
Confirmatory factor analysis of the PTCAI.

Before performing CFA, the data were checked for normality through the skewness and kurtosis method. As shown in Table 4, all skewness absolute values are less than 3 and kurtosis absolute values are less than 10, which indicates that our data generally conformed to a normal distribution (Huang, 2005). Therefore, Structure Equation Modelling via maximum likelihood method of estimation was used to perform CFA on the data using AMOS 24.0. Figure 1 shows the five-factor second-order model diagram and illustrates the factor loading coefficients of each observable variable in the model. The result indicated that Loss (0.75) and Feeling threatened (0.76) were equally important in the study participants’ negative cognitive appraisal of accidental traumas. Self-sence (0.53) was the most important positive cognitive appraisal of accidental traumas by the study participants. Positively Face (0.44) and Relationships (0.30) were somewhat behind. Models are thought to fit the data well when the chi-square/dƒratio (χ^2^/*d*ƒ) is less than 3; the comparative fit index (CFI), normed fit index (NFI), Goodness-of-fit Index (GFI), and Adjusted Goodness-of-fit Index (AGFI) are greater than 0.90; the root mean square error of approximation (RMSEA) is less than 0.08; and the Parsimony-adjusted Measures PNFI and the PCFI are greater than 0.50 (Byrne, 2016). As shown in Table 5, this model offered an excellent fit to the data, χ^2^ = 990.432; *d*ƒ = 537; χ^2^/*d*ƒ = 1.844; CFI = 0.911; IFI = 0.911; NFI = 0.901; GFI = 0.927; AGFI = 0.916; PNFI = 0.772; PCFI = 0.847; RMSEA = 0.067(90%CI, 0.053–0.082).

**Table 4:** Normality test of confirmatory factor analysis data for the PTCAI (*N* = 220).

Item	Mean	SD	Skewness	Kurtosis
1	2.89	1.20	0.02	−1.49
2	3.03	1.19	−0.13	−1.53
3	2.54	1.45	0.02	−1.31
4	2.54	1.62	−0.03	−1.44
5	2.74	1.24	0.02	−1.31
6	3.17	1.16	0.29	−1.32
7	3.19	1.45	0.27	−1.39
8	3.05	1.42	0.48	−1.47
9	2.98	1.08	−0.17	−1.46
10	3.13	1.19	0.45	−1.25
11	3.28	1.07	−0.45	−1.29
12	3.50	0.92	−0.55	−0.81
13	3.25	0.92	0.01	−1.04
14	4.23	0.61	−0.70	1.90
15	4.44	0.64	−0.96	1.91
16	4.39	0.67	−0.66	1.85
17	4.48	0.70	−0.89	1.82
19	3.52	0.88	−0.50	−0.64
20	3.20	0.80	0.34	−0.24
21	3.80	0.77	−0.42	0.01
22	4.22	0.50	0.16	0.01
24	4.63	0.56	−0.94	1.41
26	4.31	0.46	0.86	−1.28
29	3.34	1.03	−0.03	−0.99
30	3.39	1.06	−0.02	−1.17
31	3.49	0.99	−0.11	−0.82
32	3.61	0.94	−0.03	−0.72

**Table 5:** Fit index for the 5-factor 2nd order model of the PTCAI.

χ^2^/dƒ	CFI	IFI	NFI	GFI	AGFI	PNFI	PCFI	RMSEA [90%CI]
1.844	0.911	0.911	0.901	0.927	0.916	0.772	0.847	0.067 [0.053–0.082]

### 3.4 Test–retest reliability

After eliminating 2 invalid questionnaires (response time ≤ 4 min, repetitive or regular response patterns to 10 or more questions and with missing date), 18 valid questionnaires remained. The test–retest reliability results are as follows: the Positive Cognitive Appraisal Inventory, *r* = 0.822; Negative Cognitive Appraisal Inventory, *r* = 0.874; Loss, *r* = 0.865; Feeling Threatened, *r* = 0.871; Positively Face, *r* = 0.836; Self-Sense, *r* = 0.743; Relationships, *r* = 0.831 (*p* < 0.001) (see Table 6).

**Table 6:** Internal consistency reliability and test–retest reliability of the PTCAI (*N* = 220).

Dimension	Cronbach’s α	Test–retest reliability	Item number
Loss	0.869	0.865	6
Feeling Threatened	0.786	0.743	5
Negative Cognitive Appraisal	0.851	0.874	11
Positively Face	0.860	0.871	7
Self-Sence	0.743	0.831	5
Relationships	0.877	0.836	4
Positive Cognitive Appraisal	0.802	0.822	16

### 3.5 Internal consistency

With a Cronbach’s alpha of 0.802 for the Positive Cognitive Appraisal Inventory, 0.851 for the Negative Cognitive Appraisal Inventory, and 0.743–0.877 for the five dimensions, the internal consistency of the PTCAI is adequate and meets the requirement of Cronbach’s alpha >0.7. (see Table 6).

### 3.6 Concurrent validity

It was calculated how the PTCAI correlated with the SF-36, HADS, and PTGI. Loss, Feeling Threatened of the Negative Cognitive Appraisal Inventory showed significant positive associations with depression and anxiety, and significant negative associations with PTGI and SF-36 (*p* < 0.01). Loss is more closely associated with the HADS, PTGI, and SF-36, showing moderate to high correlations with them. For the Positive Cognitive Appraisal Inventory, an opposite pattern was observed. However, the Relationships simply showed low correlations with the PTGI and the Mental Health subscale of the HADS (*p* < 0.05) (see Table 7).

**Table 7:** Pearson correlations for the PTCAI with HADS, PTGI, and SF-36 (*N* = 220).

	HADS		PTGI	SF-36		
	HADS-A	HADS-D		Mental	Physical	Total
Loss	0.648^**^	0.737^**^	−0.608^**^	−0.700^**^	−0.694^**^	−0.721^**^
Feeling Threatened	0.285^**^	0.268^**^	−0.268^**^	−0.277^**^	−0.296^**^	−0.296^**^
Negative Cognitive Appraisal	0.581^**^	0.631^**^	−0.546^**^	−0.611^**^	−0.671^**^	−0.635^**^
Positively Face	−0.243^**^	−0.309^**^	0.251^**^	0.256^**^	0.304^**^	0.288^**^
Self-Sence	−0.356^**^	−0.273^**^	0.371^**^	0.428^**^	0.360^**^	0.409^**^
Relationships	−0.110	−0.112	0.171^*^	0.122^*^	0.114	0.124^*^
Positive Cognitive Appraisal	−0.391^**^	−0.388^**^	0.401^**^	0.441^**^	0.436^**^	0.453^**^

^*^Means *p*<0.05; ^**^Means *p*<0.01.

## 4 Discussion

Individuals who have experienced accidental traumatic events, physical pain, or even disability, as well as a protracted recovery process, especially those who are generally healthy, often have complex cognitive appraisals. Treated and recovering survivors of accidental traumas are able to make comprehensive evaluations, both negative and positive, from the experience of the accidental traumas through to recovery, compared to those who have just experienced the accidental traumas (Hansen, 2005). Therefore, our study developed and validated a cognitive appraisal inventory with Chinese accidental trauma survivors who were in recovery. The results confirmed the two-component structure of the Negative Cognitive Appraisal Inventory and the Positive Cognitive Appraisal Inventory and yielded five dimensions: (a) Loss, (b) Feeling Threatened, (c) Positively Face, (d) Self-Sense, and (e) Relationships, including 27 items. The five-factor structure, which accounted for 63.931% of the total variation, is closely to cognitive appraisal theory (Lazarus and Folkman, 1984) and cognitive adaptation theory (Taylor, 1983). Internal consistencies and test–retest reliabilities were satisfactory for the two subscales and five dimensions. Each item can effectively distinguish between those who have positive cognitive appraisals of accidental traumatic events and others who have negative cognitive appraisals. According to our psychometric evaluation, the PTCAI can be regarded as a valid and reliable measure of trauma-related appraisals in survivors of accidental trauma events.

The two dimensions of Loss and Feeling Threatened are similar to the Harm/Loss and Threat of CHAS (Kessler, 1998). Influenced by the uncontrollability, unpredictability, and destructiveness of the accidental traumatic events, items loading on Loss and Felling threatened reflected the survivors’ negative cognitive appraisals of traumatic accidents. Accidental traumatic events not only lead to temporary or permanent impairment of the patients’ physical functions, but sudden traumas, bloody scenes, and painful stimulations can also traumatize the patients psychologically. They also resulted in financial losses and threats to survivors’ families, jobs, and way of life. Survivors also rated the traumas as positive for them, despite the difficulties they experienced. This perspective was supported by the three dimensions of Positively Face, Self-Sense, and Relationships. Positively Face and Self-Sense are similar to Acceptance and Perceived Benefits of the ICQ (Evers et al., 2001). The PTCAI emphasized that survivors who experienced traumatic accidents were able to accept and adapt to the changes they caused, as well as perceive the benefits. The association between social support and people’s physical and mental health was demonstrated in the Relationships (Ning et al., 2017). To help survivors recover from their experiences as quickly as possible, cope better with difficulties and threats, and interpret the traumatic accidents with positive attitudes, family, friends, and medical professionals may offer them emotional and informational support. While receiving assistance from family members, relatives, and friends, survivors became closer to them and treated others in a more friendly manner.

Correlations between the PTCAI and other comparable measures of psychosocial functioning, specifically the SF-36, HADS, and PTGI, the concurrent validity was further supported. Consistent with the findings in spinal cord injury (Eaton et al., 2018), the dimensions of Loss and Felling Threatened, which reflect negative cognitive appraisals, were positively related to the HADS. This finding indicates that the higher the level of negative cognitive appraisal, the more likely they are to experience anxiety and depression. In contrast, among accidental trauma survivors, PTGI and SF-36 were negatively related to Loss and Feeling Threatened by the Negative Cognitive Appraisals. Positively Face and Self-Sense of the Positive Cognitive Appraisal Inventory noted an exactly opposite pattern. The results were consistent with the CIOQ study (Joseph et al., 2005) in which negative cognitive appraisals predicted poorer levels of posttraumatic development and quality of life, whereas positive cognitive appraisals did the reverse. This study also showed that the PTGI and the Mental Health subscale of the SF-36 were positively associated with the Relationships, showing the importance of social support for posttraumatic growth and mental health. Unhealthy relationships can make it difficult for survivors to fully accept unintentionally terrible experiences (Ning et al., 2017).

There are areas in which this measure requires further exploration. The data sets presented here predominantly comprise participants exposed to accidental traumas that did not last for longer than a few minutes and affected a few people (e.g., MVAs). Individuals exposed to more enduring and widely devastating traumatic stressors, such as wars or natural disasters, may appraise their experiences in a in a more negative or positive way. Furthermore, the usefulness of the PTCAI has not been examined in people exposed to sexual abuse. This study inevitably has limitations. Firstly, our sampling method prevented us from knowing how representative our samples were of accidental trauma survivors. Only accidental trauma survivors in China make up our samples. However, given that racial/ethnic differences have been observed on psychological measures among trauma survivors with PTSD (Ruglass et al., 2020), the measurement properties of the PTCAI are likely to be influenced by the racial/ethnic disparities of the posttraumatic survivors, and this convenience sampling method is not ideal for psychometric studies. Secondly, the association between the personalities of survivors and the duration of the stressful interaction with posttraumatic cognitive assessments is not covered in the current study, but could be explored in future studies. Additionally, the PTCAI was developed and validated in Chinese before being published in English. Back-translation and additional validation in Western populations are required in order to assess its psychometric features. Future studies will need to address these issues if this measure is to have broader usefulness.

## Data availability statement

The raw data supporting the conclusions of this article will be made available by the authors, without undue reservation.

## Ethics statement

The studies involving humans were approved by Medical Ethics Committee of Shandong First Medical University. The studies were conducted in accordance with the local legislation and institutional requirements. The participants provided their written informed consent to participate in this study. Written informed consent was obtained from the individual(s) for the publication of any potentially identifiable images or data included in this article.

## Author contributions

AZ, WZ, and WY conceived and designed the analysis, collected the data, performed the analysis, and wrote the manuscript. BW and QD wrote sections of the manuscript. All authors contributed to the article and approved the submitted version.
