# Supplementary material for: The posttraumatic cognitive appraisal inventory (PTCAI): development and validation
Source: Front Psychol. 2023 Dec 22;14:1224984. doi: 10.3389/fpsyg.2023.1224984 (PMC10771330; doi:10.3389/fpsyg.2023.1224984)
Supplement: Supplementary file 1 [file Data_Sheet_1.docx]

Supplementary Material

The Posttraumatic Cognitive Appraisal Inventory (PTCAI): Development and Validation

Wenyue Zhang^1^, Wenjing Yu^2^, Baojian Wei^1^, Qianni Dong^1^, Aihua Zhang^1^*

^1^School of Nursing, Shandong First Medical University& Shandong Academy of Medical

Sciences, Tai'an, Shandong, China.

^2^Tai'an Municipal Hospital, Tai'an, Shandong, China.

**Equal contributions:** Wenyue Zhang 1† and Wenjing Yu 2† contributed equally to this work and share first authorship.

*** Correspondence:** Aihua Zhang*, School of Nursing, Shandong First Medical University & Shandong Academy of Medical Sciences, Tai'an, Shandong, China.
Email: [zhangah818@163.com](mailto:zhangah818@163.com)

# Supplementary Figures


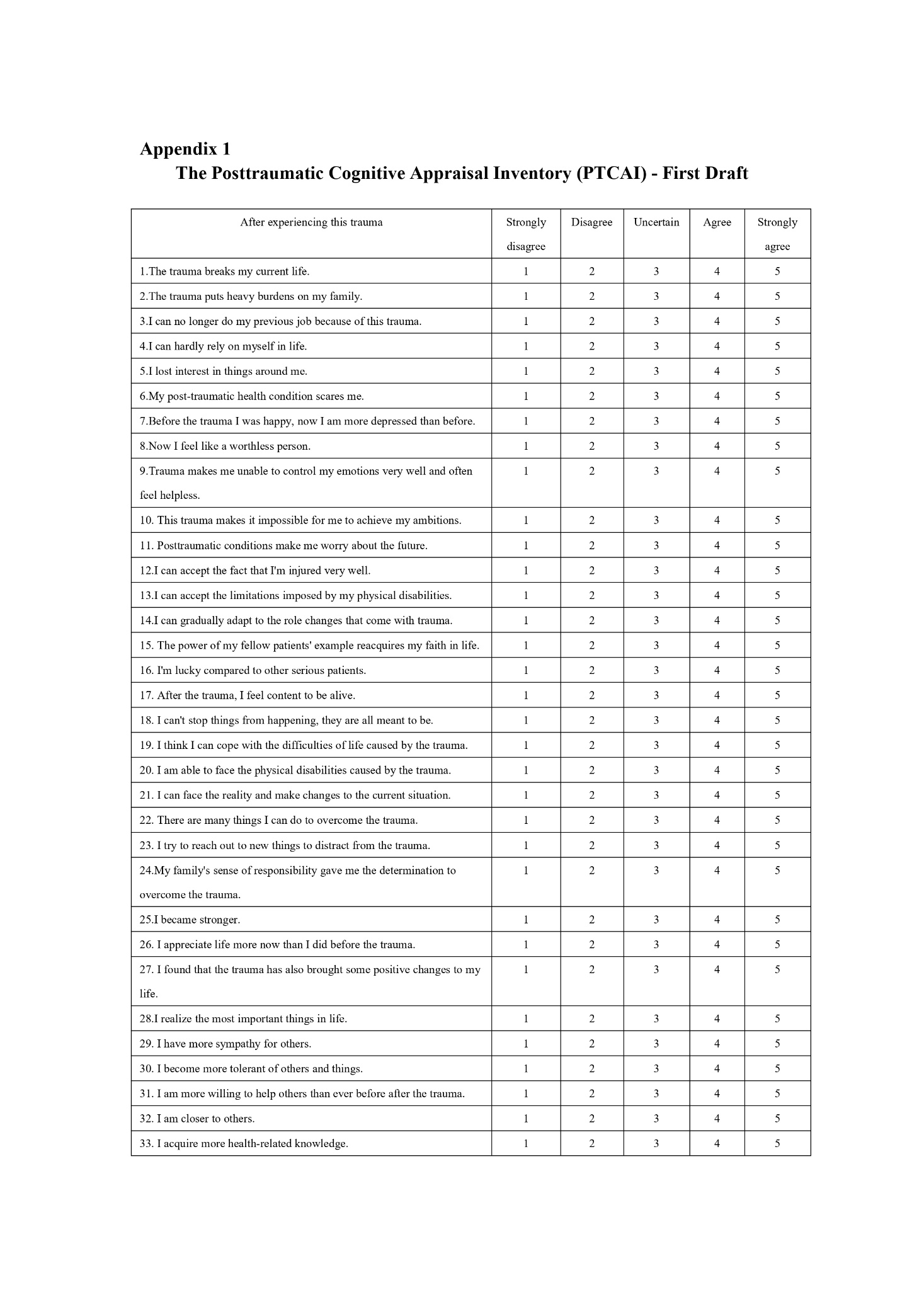


**Supplementary Figure 1.** The Posttraumatic Cognitive Appraisal Inventory (PTCAI) – First Draft


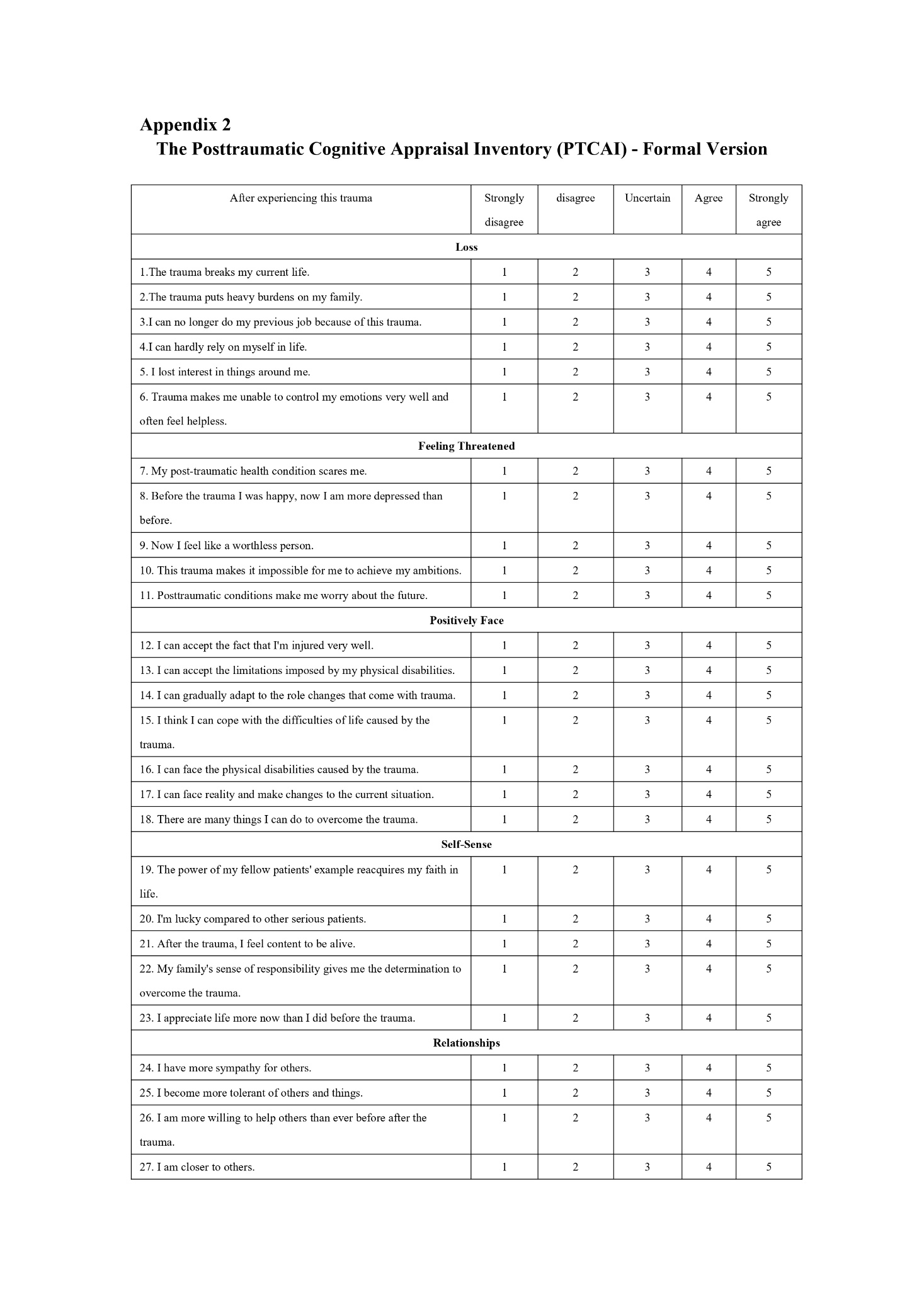
 **Supplementary Figure 2.** The Posttraumatic Cognitive Appraisal Inventory (PTCAI) – Formal Version
